# Increased Incidence of Obesity in Children and Adolescents Post-COVID-19 Pandemic: A Review Article

**DOI:** 10.7759/cureus.29348

**Published:** 2022-09-20

**Authors:** Sushmita Jha, Ashok M Mehendale

**Affiliations:** 1 Public Health, Jawaharlal Nehru Medical College, Datta Meghe Institute of Medical Sciences, Wardha, IND; 2 Preventive Medicine, Jawaharlal Nehru Medical College, Datta Meghe Institute of Medical Sciences, Wardha, IND

**Keywords:** children, adolescents, covid -19, coronavirus pandemic, overweight, obesity

## Abstract

The recent coronavirus 2019 (COVID-19) pandemic has immensely impacted all classes of society, but the effects on children and adolescents are much more pronounced than on others. While obesity and its comorbidities in children and adolescents have always been a concern, the COVID-19 pandemic has proven to be one of the leading causes of health problems in children and adolescents worldwide, leading to various complications. Hence, understanding its long-term sequelae is of utmost importance. The role of physicians in family counseling, nutrition counseling, and diet education is vital in maintaining a healthy lifestyle. The BMI (body mass index) measurements and retrospective cohort studies of various individuals are useful for the pertinent research. During the pandemic, social isolation, staying at home, increased screen time due to online classes, reduced outdoor activities, and more snacking are some of the contributing factors that have increased the prevalence of obesity and further morbidities associated with it. Multiple studies and guidelines are available for combating these issues; still, an increasing number of such cases have been encountered in routine outpatient department (OPD) practice. As opposed to specific infectious illnesses, obesity and its comorbidities are non-infectious, and a slow-growing silent risk; hence parents approach the pediatrician quite late in the disease process. With the emergence of the COVID-19 pandemic, every aspect of our life has entered a more virtual domain and is no longer confined to a mere physical sphere. This sudden shift to virtual online classes has significantly impacted children and adolescents by decreasing their physical activities and social interactions in schools. This has even led to increased use of social media and mobile phone games by children and adolescents, a grave concern for parents, pediatricians, and epidemiologists. A more detailed assessment and multidisciplinary approach might benefit in dealing with the management of this emerging issue. Gaining enhanced clarity by establishing more guidelines can help physicians as well as parents in the management of this critical issue.

## Introduction and background

Obesity is a complex disease that involves an excessive amount of body fat. It is both a medical and a cosmetic concern, increasing the risk for various other complex disorders and health issues [1]. The incidence of obesity has increased worldwide and is now a major public health challenge, especially among young people. Obesity in children and adolescents has become a pandemic and is currently one of the most common health issues [2]. Body mass index (BMI), expressed as weight in kilograms divided by height in meters square (kg/m^2^), is commonly used for grading obesity among adults, children, and adolescents. Obesity in childhood and adolescence increases the risk for hypertension, mental health issues, and poor achievements at school in young children. It may lead them to suffer from obesity throughout life, cardiovascular diseases, type 2 diabetes mellitus, and other diseases that can lead to complications or even death [3]. Cases of coronavirus disease 2019 (COVID-19) have spread worldwide over the past two and a half years, leading to global lockdown, social distancing, home isolation, and the closing of schools [4]. This has caused an increase in screen time and decreased outdoor and physical exercise, causing an increase in weight, lethargy, decreased social interaction and development, weak eyesight, as well as obesity, and various other morbidities in children and adolescents. As more than 80% of children were affected by the closing of schools and disruption in their lives, there is a risk of deterioration of child health and an increase in cases of childhood obesity [5].

Increased weight gain may lead to behavioral changes like an unbalanced diet and unhealthy eating habits, inactivity, and improper sleep, and children may even suffer from depression and insomnia. This has been attributed to the increased use of mobile phones and computers by children, especially school-going children, during the pandemic, as it was challenging to organize offline classes due to measures such as lockdowns and isolation [6]. The sudden and prolonged continuous stress of quarantine and the pandemic may lead to a negative psychological effect on almost all children and their families [7]. This might lead to unhealthy food habits because of stressed eating and cause obesity in children [8]. The lockdown has led to financial losses, and the pandemic has caused difficulty among families in terms of affording healthy food items.

In this review, we aim to bring further attention to the harmful effects of the COVID-19 pandemic and analyze the inter-relationship between the COVID-19 pandemic and obesity in children and adolescents by assessing changes in their BMI during the pandemic. We also intend to provide recommendations on life changes to prevent or manage obesity and obesity-related comorbidities among our future generation of children and adolescents.

## Review

Obesity is a complex disease that involves an excessive amount of body fat and it is not just a concern from a cosmetic point of view. It is a major risk factor for various comorbidities and complications [1]. Childhood obesity incurs social, economic, and epidemiologic costs, and leads to a decrease in economic productivity; an increase in obesity cases is a big burden on healthcare systems [2]. Due to lockdowns during the COVID-19 pandemic, there was a huge increase in the cases of obesity, especially in children, adolescents, and young adults.

Increase in the incidence of obesity during the COVID-19 pandemic

According to some studies, the cases of obesity and its related comorbidities increased significantly during the COVID-19 pandemic. Before 2019, there were fewer cases of obesity all around as compared to now. This is primarily because of changes in the diet of children and adolescents. The pandemic caused a lot of changes in food habits and a decrease in food availability, which is a significant risk factor for disruption in a child's physical, mental, and social well-being. Many children of poor socioeconomic status depend on the meals provided by their school for a proper and healthy balanced meal with adequate nutrition, which got disrupted because of the lockdowns during the pandemic [9]. A study across India during this pandemic revealed that there is a severe deterioration in eating patterns and proper food consumption by many children and adolescents, along with eating unhealthy food such as snacks and eating more than necessary [10]. The closing of schools led to decreased physical activity, which affected the mental health of children. The closing of schools increased children's sedentary time from five to 8-10 hours due to online classes. Thus, less time was spent outside doing any physical, social, or productive activity by children. This decreased intensity of doing any physical work, completing homework, or studying. Along with these, sedentary behavior, decreased social interaction, fear of attending canceled exams, and social distancing during the closing of schools also caused depression and stress. This increase in anxiety may lead to overeating, causing obesity, decreased social interactions and further psychological issues, and low self-esteem in the future.

Increase in childhood obesity rates and its long-term consequences

There is much evidence of increased obesity rates in all age groups during the pandemic. If not prevented or adequately managed, obesity in children may cause various consequences such as the risk of mental health disorders, heart diseases, diabetes, cancer, and other diseases. This increased risk of associated comorbidities due to obesity may cause death at a young age, especially in people of low socioeconomic status [11]. Table 1 shows the prevalence of obesity and overweight in various age groups and gender among children and adolescents. We observed that the prevalence of obesity is more in the age group of 16-18 years and more common in males than in females [12]. 

**Table 1:** Prevalence of obesity and overweight in children and adolescents with type 1 diabetes mellitus* *[12]

Age group/gender	Overweight (%)	Obesity (%)
5-9 years	18	18
10-15 years	15	17
16-18 years	25	21
Male	16	20
Female	19	16
Total group	17	18

Studies that can be done to ascertain obesity in a population group

There are two types of studies that can be performed for assessing the incidence of obesity nationwide.

Cohort Studies

A cohort study is a research study that compares a particular outcome (such as obesity) in groups of individuals who are similar in symptoms but differ from each other by a specific characteristic (e.g., obesity in young people leads to more complications compared to elders) [13].

Body Mass Index Studies

BMI is derived from the mass and height of a person. The BMI is defined as the body mass (in kg) divided by the square of the body height (m²). It gives us the grade of obesity. Table 2 shows the various grades according to the BMI of an individual. The normal range of BMI is 18.5-24.9. A BMI of less than 18.5 is considered underweight and more than 24.9 is considered overweight. A BMI of more than 30 is considered obese. There are three grades of obesity: classes one, two, and three [14].

**Table 2:** Grade of obesity according to BMI BMI: body mass index

BMI (kg/m²)	Grade
<18.4	Underweight
18.5-24.9	Normal range
25.0-29.9	Overweight (pre-obese)
30.0-34.9	Obese (class I)
35.0-39.9	Obese (class II)
>40.0	Obese (class III)

Children with obesity affected by COVID-19

COVID-19 is usually asymptomatic in almost all children and adolescents or mostly remains undiagnosed with mild symptoms. Still, many children and teenagers have been affected by this pandemic in the past year, increasing the number of cases [15]. Those who suffered from severe infection by the coronavirus had symptoms such as low oxygen saturation, dyspnea, and acute respiratory distress syndrome (ARDS); some also had a corona-associated multisystem inflammatory syndrome (MIS-C) [16]. MIS-C is a severe problem leading to multiorgan failures, cardiovascular shock, and other complications. It is also known as pediatric inflammatory multisystem syndrome (PIMS) as it is more common in the pediatric age group, and it mostly occurs after the COVID-19 infection. It is an uncommon but severe disease [17]. Many studies and reviews have shown that obese children had a high risk for both MIS-C and various other respiratory disorders as complications of infection by coronaviruses compared to those who are healthy [18]. A more structured and proper systematic study and surveillance should be conducted in order to properly understand the effects of the COVID-19 pandemic on children and to ascertain which age group is at the highest risk for MIS-C and other severe illnesses.

Rise of obesity cases post-COVID-19 pandemic

In the year 2019, which was before the COVID-19 pandemic, the cases of obesity around the globe were normal and controlled. But due to lockdowns, home isolation, and online classes during the pandemic, cases of obesity increased in children and adolescents by the end of the year 2021. At this rate, achieving the goal of a healthy community will be challenging. According to a study conducted among adolescents, during the initial three to four weeks of lockdown, exercise time decreased, and eating of unhealthy junk food increased. It was also found that obese people were more likely to get affected by COVID-19, as obese people are three times more prone to severe long-term complications, which include dyspnea, pain in the chest, and decreased immunity. It was also concluded that countries with more obese populations had higher mortality rates due to COVID-19 [11]. This rise in cases of obesity is a matter of concern and should be prioritized since the most affected population consists of children and adolescents.

Complications of obesity in children and adolescents

**Figure 1:**
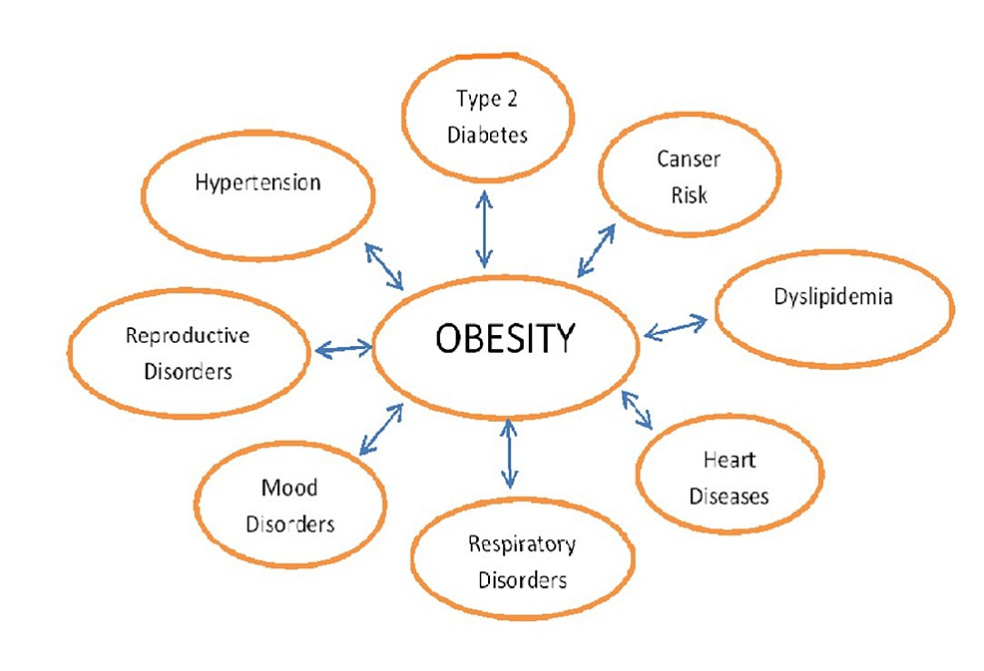
Flowchart depicting complications of obesity

Due to increased obesity cases in children, complications related to health deterioration have become more common [19]. The various complications of obesity and overweight are as follows: type 2 diabetes mellitus, heart diseases and stroke, sleep apnea, digestive problems (heartburn, gallbladder problems, and liver diseases), certain cancers (like cancer of the ovary, colon, endometrium, liver, kidney, uterus, rectum, breast, prostate, pancreas, cervix, gallbladder, and esophagus), osteoarthritis, and severe COVID-19 symptoms [1]. Some of them are depicted in a flowchart shown in Figure 1. The first one is a change in glucose metabolism and its abnormalities, which can cause type 2 diabetes mellitus. It can lead to several health issues in children and adolescents and, if not prevented or treated correctly, may lead to adult morbidities and even mortality in old age [20]. Obesity during childhood can cause metabolic and cardiovascular complications usually related to increased levels of insulin in the blood and its resistance and other associated complications of obesity [21]. It is challenging yet crucial for a physician or epidemiologist to identify children and adolescents at greater risk of being overweight or obese. Till now, the steps taken to prevent weight gain and initiate weight loss in children and adolescents have not been successful, and we should provide adequate resources with regular monitoring and follow-up for the same. This leads to a lot of confusion regarding the application of these limited resources and which age group of obese children will get the most benefit from the behavioral change or therapeutic interventions [22].

The increasing rate and severity of obesity cases in children have Ied to an increase in another pediatric disease, type 2 diabetes mellitus, as obesity affects glucose metabolism. The risk for type 2 diabetes mellitus in children and adolescents has increased immensely worldwide due to the increase in obesity [23]. Still, controlling obesity and regulating blood sugar levels can prevent diabetic complications of obesity; it can be done by either lifestyle changes or therapeutic measures [24]. All these factors led to an understanding that diabetes mellitus, primarily type 2, is most commonly associated with obese individuals and should be a matter of concern for parents and doctors.

Obesity is a significant risk factor for CVD, dyslipidemia, type 2 diabetes, hypertension, arrhythmias, and sleep disorders (Figure 1). According to recent studies, cardiovascular risk factors are primarily independent of BMI. These can be prevented by lifestyle changes [25]. CVD is a significant risk factor for obesity, and these risks are prevalent in old age with prolonged obesity cases. Obesity-associated health service use and medical costs, as well as the diseases related to it, have increased unexpectedly and will continue to rise further [26]. Genetics plays a vital role in the epidemic and development of obesity, which has led to the conclusion that obese children with a genetic history of CVD are more prone to CVD [27]. Any CVD in children or adolescents is a significant sign of danger, as young children with CVD are more prone to mortality; hence, an obese child should be monitored for cardiovascular complications.

Steps to be taken for the prevention and treatment of obesity

Prevention of any disease's comorbidities and complications is essential for a healthy lifestyle. As compared to the pre-pandemic era, doctors as well as patients and their parents, along with epidemiologists, are much more aware of obesity, its comorbidities, its complications, and prevention and treatment of the same now. A few steps that can be taken to prevent obesity in children are described in the following paragraphs.

The first and foremost step involves promoting a healthy lifestyle. It can be followed by adopting a properly balanced diet with an adequate amount of macronutrients and micronutrients, having a reasonable weight goal, and trying to maintain that. Family should work together with all the members to help overweight individuals and should not differentiate them from others. They should change family eating habits and physical activities, plan daily timings for every meal and manage snack timings; parents can also make a food pyramid for young children to make it enjoyable for children to follow a proper food diet and eating habits [28].

Developing healthy eating habits is also an important step to be followed. To create healthy eating habits in children, an adequate amount of vegetables and fruits should be given. Low-fat or non-fat milk with less amount of butter and saturated fatty acids (SFAs) should be included in the diet, along with a proper amount of proteins such as meats, fish, peas, and cereals; mid-meals snacks should be avoided to maintain a proper and healthy balanced meal three times a day. They should also avoid late-night snacks, drink plenty of water, and should decrease their consumption of alcohol, sugary drinks, and saturated fat [29].

It is very important to engage in an adequate and good amount of physical activity as it is essential to decrease weight and improve lifestyle. Steps to be taken during the lockdown and home isolation to increase physical activity are as follows: parents should limit the unnecessary screen time of their children, such as decreasing the use of televisions, computers, and mobile phones, and children should take small breaks of about 30 minutes between using the screens for too long, like about two to three hours, and make sure that they stretch, walk, or stand during these breaks. Parents should encourage children to do physical exercise and other activities. They should insist that children perform activities that will promote a healthy and exciting way of losing weight [30]. They can create fun toys and introduce fun techniques to children to encourage them to do more physical activities and decrease their screen time by distracting them when they are using mobile phones and computers for playing games. Activities such as making flashcards regarding study subjects and hiding them to ask them to find them like a treasure hunt and then answering the question in the flashcard can be done. This will help them in learning as well as in doing some physical work.

Some medications and weight-loss surgeries are also helpful in some cases. Some of the weight management medications are orlistat, phentermine-topiramate, naltrexone-bupropion, and liraglutide. There are some medicines that decrease the desire to eat, such as phentermine, benzphetamine, diethylpropion, and phendimetrazine [31]. A few types of weight-loss surgeries are as follows: gastric bypass surgery, gastrostomy, and adjustable gastric banding [32].

The role of the family in the prevention of obesity is also very important. All family members should influence and encourage the obese child and their parents to control their weight gain and to take treatment and stick to it and help them with changes in their lifestyle. According to the National Institute of Health and Care Excellence (NICE) obesity management guidelines, the patient's family members should encourage and support them in managing their weight according to the weight management program for success [33]. An approach involving the whole family is essential, especially in addressing childhood obesity, and all the programs made for the family should be promoted and encouraged [34].

Role of the government in reducing obesity: governments play an important role in raising awareness regarding any public health issue and its prevention. They should initiate a protocol for providing information about nutrition and the amount of fat and calories in all different types of food items, especially fast foods, at all restaurants. There should be a ban on selling junk and unhealthy foods in and around schools. They should encourage the supply of fresh fruits, vegetables, and healthy foods to poor people. They should provide education about healthy eating and physical exercises for a better lifestyle at schools [35]. The five A's that are adopted by the government for the management of obesity are as follows: ask, assess, advise, agree, and assist.

Based on our literature review, we have concluded that obesity cases have increased significantly due to the pandemic, leading to various complications, and it is very important to prevent further cases and complications in children and adolescents (Table 3).

**Table 3:** Various studies assessing obesity in children and adolescents due to the COVID-19 pandemic

Author	Title	Source	Findings
Di Cesare et al. (2002) [2]	The Epidemiological Burden of Obesity in Childhood: A Worldwide Epidemic Requiring Urgent Action	BioMed Central (BMC) Medicine	The incidence of obesity has increased worldwide and is now a public health challenge, especially among young people. Obesity in children and adolescents has become a pandemic and is currently one of the most common health issues
An (2002) [3]	Projecting the Impact of the Coronavirus Disease 2019 Pandemic on Childhood Obesity in the United States: A Microsimulation Model	Science Direct	BMI is commonly used in classifying obesity among adults, children, and adolescents. Obesity in childhood and adolescence increases the risk of various complications in young children. It may cause obesity throughout life, cardiovascular diseases, type 2 diabetes mellitus, and other diseases that can even lead to death
Guo et al. (2021) [6]	Physical Activity, Screen Exposure, and Sleep Among Students During the Pandemic of COVID-19	Scientific Reports	Increased weight gain may lead to behavioral changes like an unbalanced diet and unhealthy eating habits, inactivity, and improper sleep, and may even lead to children suffering from depression and insomnia. This all happened due to the increased use of mobile phones and computers by children, especially school-going children, during the pandemic, as it was challenging to organize offline classes due to measures such as isolation
Rasheed (2017) [8]	Stress-Associated Eating Leads to Obesity	Nature Food	Lockdown and home isolation have led to decreased concentration among students in studies and decreased knowledge and marks, which might lead to unhealthy food habits because of stressed eating and cause obesity in children
Carducci et al. (2021) [9]	Food Systems, Diet, and Nutrition in the Wake of COVID-19	Intermediate Journal of Health Sciences	Cases of obesity and its related comorbidities increased significantly during the COVID-19 pandemic. This is primarily because of changes in the diet of children and adolescents. Many children of poor socioeconomic status depend upon the meals provided by their school for a proper and healthy balanced meal with adequate nutrition, which got disrupted because of the lockdowns during the pandemic
Dietz (1998) [20]	Health Consequences of Obesity in Youth: Childhood Predictors of Adult Disease	PubMed	The main complications of obesity are changes in glucose metabolism and its abnormalities, which can cause type 2 diabetes mellitus. It can cause several health issues in children and adolescents and, if not prevented or treated correctly, may lead to adult morbidities and even mortality in old age
Weiss and Kaufman (2008) [21]	Metabolic Complications of Childhood Obesity: Identifying and Mitigating the Risk	Diabetes Care	Obesity during childhood can cause metabolic and cardiovascular complications usually related to increased levels of insulin in the blood and its resistance and other associated complications of obesity
Polfuss et al. (2020) [30]	Childhood Obesity: Evidence-Based Guidelines for Clinical Practices – Part One	Journal of Paediatric Health Care	Prevention of obesity is very important and involves doing an adequate and good number of physical activities as it is essential to decrease weight and improve lifestyle. There are various steps that can be taken during the lockdown and home isolation to increase physical activity, which is mentioned in the article

## Conclusions

Obesity is a complex disease that involves an excess of body fat. It is both a medical and a cosmetic concern, increasing the risk for various other complex disorders and health issues. Its incidence has increased during the COVID-19 pandemic across the world, which led to a lot of consequences and complications. We discussed two methods to measure obesity: BMI and retrospective cohort studies. It was found that obesity cases severely increased during the pandemic, that is, in the last 2.5 years. This increase in the cases of obesity and its comorbidities has led to various complications. The rise in the incidence of obesity, especially in children and adolescents, was due to decreased physical activities, stress, overeating, and staying at home almost all the time due to global lockdowns, home isolation, and the closure of schools and online classes. We also discussed the complications due to prolonged untreated obesity in children. We also dwelled on the role of the patients, their parents, their family, the doctors, the epidemiologists, and the government in the prevention of obesity and treatment. Now, even doctors, the child, and their parents are fully aware of obesity and its effects, the measures taken to prevent obesity in children, and the treatments to prevent complications. The association between the COVID-19 pandemic and an increase in obesity cases and its complications has been well-documented. Finally, we can conclude that the COVID-19 pandemic has led to a rise in the cases of obesity in children and adolescents, which is a risk factor for many severe comorbidities and can lead to many complications like CVDs, fatigue, type 2 diabetes mellitus, and many more. Hence, it is essential to prevent its prevalence and manage obese children appropriately to avoid future complications.
